# Dreaming About Travel: A Pinterest Netnography

**DOI:** 10.1007/978-3-030-65785-7_23

**Published:** 2020-11-28

**Authors:** Ulrike Gretzel

**Affiliations:** 1 Department for Informatics, Technical University of Munich, Garching bei München, Bayern Germany; 2 Smart Tourism Education Platform (STEP) College of Hotel and Tourism Management, Kyung Hee University, Seoul, Korea (Republic of); 3 Department of Tourism and Service Management, MODUL University Vienna, Vienna, Wien Austria; University of Southern California, Los Angeles, USA

**Keywords:** Ongoing information search, Dreaming phase, Netnography

## Abstract

Ongoing travel information search remains under-examined in general, and specifically in terms of social media use. Understanding how visual social media platforms inspire travel dreams is increasingly pertinent as visual contents gain in importance. This is especially relevant when travel is restricted, such as during the COVID-19 pandemic. Pinterest seems to be ideally suited for supporting ongoing search but has been rarely used as a data source in e-tourism research. This paper uses a netnographic approach to explore travel-related Pinterest data. From a methodological perspective, it finds that the platform is suitable for informing ongoing travel information search research but points to potential methodological challenges. As a theoretical contribution, it highlights the popularity of capturing travel dreams through Pinterest boards and illustrates the affective labor users put into their collections of travel dreams. The paper concludes with implications for tourism marketing and recommender system design.

## Introduction

“Dreaming about travel” as a stage in the travel process that involves looking for inspiration is generally recognized as an important phase because of the hedonic value to consumers and the persuasive potential for tourism marketers. In practice, it is often very narrowly defined as information search without firm travel plans and as starting when travelers think of their next vacation [1]. As such, it initiates a trip cycle or travel customer journey [2]. From a theoretical perspective, dreaming about travel is a more loosely defined concept that involves ongoing information search without any relation to a specific trip. It entails building up a general knowledge base for unspecified future travel and can last for a lifetime [3]. Visual contents are particularly important in the dreaming phase [2] and the visual turn in social media contents and use [4] seems to indicate that social media have become even more suitable for supporting travelers in imagining their dream vacations. Despite the theoretical need to understand how consumers construct their travel dreams and the practical necessity to identify what types of information sources and channels inform the process, little is currently known about this “dreaming” phase.

The Washington Post [5] reports that even, or maybe especially, during the travel restrictions imposed by the COVID-19 pandemic, Internet users have been busy looking for inspirational content, including travel-related information. It mentions Pinterest as one of the platforms where consumers look for travel ideas. Indeed, Pinterest seems to be ideally suited for catering to ongoing information needs as it allows users to create extensive curations of contents they find relevant or motivating. Since its inception, the platform has included a default board called “Places I’d Like to Go” to which users can pin travel contents. Surprisingly, little is known about Pinterest as a source of travel inspiration. The research in this paper therefore seeks to explore Pinterest data and its suitability as a data source for research on ongoing information search. It also aims at deriving preliminary insights on the kinds of ongoing searches Pinterest users engage in and the patterns that emerge from their pinning activities.

## Literature Review

### Ongoing Information Search

While information search has been a central topic in tourism research [6], theoretical progress on the role of technology, and especially social media, in supporting travel information search has been slow [7]. This is particularly true for ongoing information search, which has received very little attention in the literature. Although mentioned in traditional travel information search models [3], its conceptualization in online contexts and its measurement have received little attention [8, 9]. It is typically buried in the “prior knowledge” category when discussing information search [10], without considering how it comes about and what role social media play in its construction. Alternatively, it is explained away with enduring involvement in travel and mixed up with activities that express it, such as subscribing to travel magazines. Social media represent a unique opportunity to explore ongoing travel information search as they make related processes explicit and provide records of the “travel dreams” users construct when engaging with contents. As explained above, Pinterest seems to be particularly important for travelers as an external memory device that supports ongoing searches.

### Pinterest

Pinterest was founded in 2010 and has since grown to over 400 million monthly active users [11]. It is an image sharing platform which also features an AI-powered visual search engine that allows users to discover contents. Users, so-called Pinners, curate online contents in the form of pins that are either directly uploaded to the platform or link to contents available on the Internet. As such, it differs significantly from other platforms like Instagram or Facebook that allow for the sharing of contents but not their thematic organization. These pins are organized into boards that represent different topic areas. The pins consist of an image with a short label and an indication of whether the pin was created by the user or repinned from other Pinterest accounts. Such repinning is actively encouraged by the platform through daily emails that make suggestions and by featuring “related ideas” within the boards themselves. The platform currently houses over 200 billion pins organized into 4 billion boards [12].

While the platform is global, users from the United States still dominate [13]. Almost 70% of the users are between 18 and 49 years old [14] and the majority of users (71%) are female [12]. In the United States, Pinterest reaches 83% of women aged 25–54; this group makes 80% of the buying decisions in US households [15]. Importantly, 89% of US Pinners use Pinterest for inspiration in their path to purchase [15].

Compared to other social media platforms, tourism-related research on Pinterest or using Pinterest data is almost non-existent. Existing studies explored the adoption of Pinterest for destination marketing [16] and analyzed the destination image of Japanese cities using Pinterest data [17]. Research on pins that show celebrities at airports is also tangentially related to tourism [18]. More recent studies conducted an automated analysis of Thailand’s destination image using Pinterest [19] and analyzed the heritage destination images shared on the platform [20]. Overall, the potential of Pinterest to inform tourism research remains grossly underexplored.

## Methodology

Netnography is a qualitative research method that allows for in-depth, contextual understanding of data derived from websites, social media or mobile phone applications [21]. Such deep understanding of Pinterest as a platform and of its users and uses in the context of ongoing travel information search was deemed necessary to inform this research. Netnography has been used extensively in tourism research to study a wide range of phenomena [22, 23] but seems underused in the e-tourism field, despite the “natural fit” pointed out in [24]. In contrast to other qualitative methods for digital data collection and analysis, netnography provides an explicit set of operational procedures [21]. These are organized into six procedural movements related to finding research questions (initiation), collecting data (investigation, interaction, immersion), analyzing and interpreting data (integration) and communicating the results (incarnation). The initiation phase includes ethical considerations. This research only collected small-scale, publicly available data and used pseudonyms when referring to specific Pinterest users.

### Data Collection

Two netnographic data collection operations (investigation and immersion) were combined in this research. Investigation involves the systematic collection of existing digital data while immersion describes the researcher’s “inhabitation” [21, p. 140] of relevant digital spaces. The data site for this research was pre-determined because of the theoretical and methodological interest in Pinterest as a platform that specifically caters to ongoing travel information search.

#### Immersion.

Immersion is a signature move for netnography as it fosters engagement with the data in context. Rather than being focused on quantity, length or intensity of participation, immersion in netnographic research is data-centric and involves the search for deep, insightful data. According to [21], immersion encompasses reconnoitering, recording, researching and reflecting and has sense-making as its ultimate goal. Data is recorded in the form of an immersion journal to support the curation of insights.

**Fig. 1.**
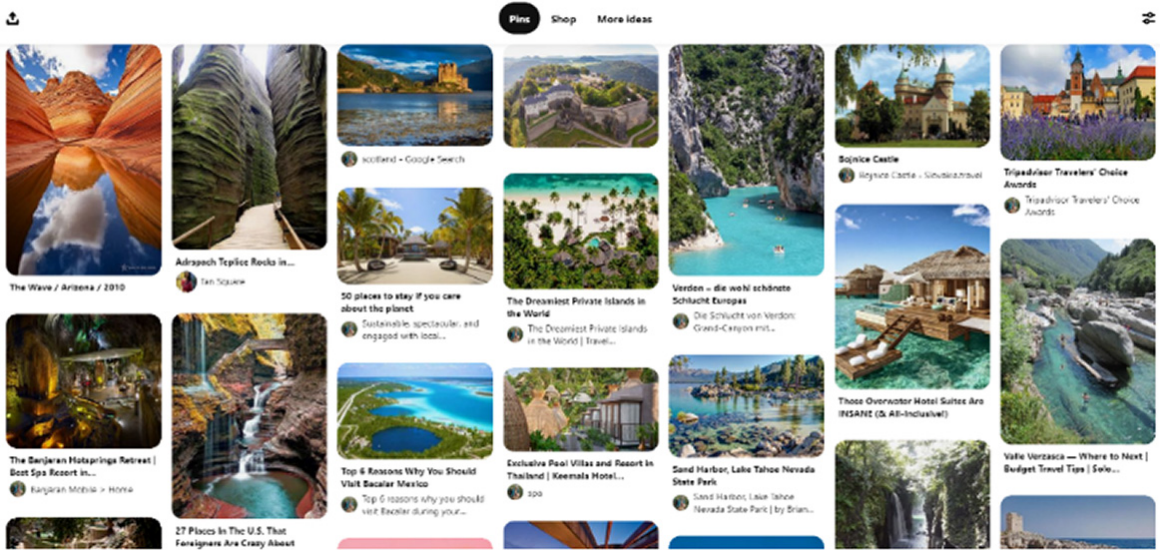
The author’s “Places I’d Like to Go” Pinterest board.

The author signed up for Pinterest shortly after it became publicly available in the United States and has been using the default “Places I’d Like to Go” board ever since (Fig. 1). Thus, the netnographic immersion could build on a decade’s worth of experience with the platform, its notification emails and the activities of other users. The exposure to Pinterest was intensified for a four-week period in 2020. It focused on exploring travel-related boards of other Pinterest users and noting interesting patterns that emerged in terms of structure as well as contents.

#### Investigation.

The standardized “Places I’d Like to Go” boards served as the sole data site for the investigative part of the netnography. These boards were accessed through a simple board-based search on Pinterest using the exact title. Pinterest displays thousands of boards for this search but does not provide an exact number of search results, neither is the ranking of the results clear. The order of the boards also changes somewhat when the search is repeated. While this is not particularly problematic but still annoying for qualitative data collection, it constitutes a challenge for systematic data sampling. To allow for exploration of the potential for automated analysis, the first 100 boards that appeared in the search results and represented individual users rather than corporate accounts were selected. The search was conducted in September 2020.

For these boards, the URL of the board, the number of followers, the number of pins, and the username of the board creator were manually captured in a spreadsheet as these were the types of information readily available for every board. The display of the pins changes dynamically, and is interspersed with advertisements. Also, pins have a standardized width but can differ substantially in length and can span several rows. Only the top row does not change and was therefore used for sampling purposes. This row contains a maximum of 7 pins but often only displays 6 images as some boards have pin suggestions in the first pin position. Even though the boards are clearly travel-related, not all pins are. Only pins that featured travel-related contents were considered. Pinning mistakes happen quite frequently as Pinterest automatically selects the most likely board/section to which the pin belongs. This means that inconsistent data structure and data quality are an issue [25]. A total of 609 pins were considered for analysis.

The coding of pin contents was conducted by the author and involved simple determination of the presence or absence of image features. No distinction was made between videos, composite images and simple still images as the image modality does not necessarily affect manual coding; however, the different types of image contents could be problematic for automated analysis.

Location of the displayed image was coded at the continent level. The coding also involved the identification of popular image categories. The emphasis was on features that could be easily identified by visual search engines/image classification algorithms but also hint at the types of vacations Pinners are dreaming about. A group of 10 boards identified during the immersion phase was used to derive these categories and resulted in eight features that were coded: 1) water (ocean, lakes, rivers, pools, waterfalls, etc.); 2) mountains; 3) beaches; 4) cityscapes; 5) snow & ice; 6) churches or temples; 7) food, and 8) animals. This coding scheme was then applied to the main sample of pins.

### Data Analysis

Netnographic analysis of data is flexible in that it allows for deductive, inductive and abductive reasoning as well as telescopic and microscopic levels of analysis [21]. The data analysis for this research was inductive and occurred at both levels. Structural patterns provide important contextual information that allow for interpretation of detailed findings. Structural analysis was also necessary to inform methodological considerations regarding the suitability of Pinterest data for automated forms of data analysis. The structure and content of the 100 Pinterest boards were explored through descriptive statistics to reveal such larger patterns in the data. The analysis was interwoven with insights from the immersion data. At the microscopic level, the analysis involved in-depth exploration of individual boards and identification of interesting patterns within and across these boards. During the analysis, a total of four cases were identified as particularly illustrative of the diversity of board structures and contents.

## Findings

The findings illustrate the richness of data about ongoing travel search readily available through Pinterest, demonstrate the flexibility of netnography in terms of zooming in and out of data to explore insights at different levels, discuss how the platform shapes travel dreams, and present commonalities and differences across Pinners and boards.

### Descriptive Analysis of Pinterest Boards

As mentioned above, 100 “Places I’d Like to Go” boards on Pinterest were analyzed in terms of their characteristics and top row contents. The results of the descriptive analysis indicate that there is diversity in the boards but also suggest some interesting trends. They further hint at methodological challenges for automated types of analysis.

#### Creators.

The creators of these boards were overwhelmingly female (94%); only 5% were male and one creator’s gender could not be identified. In general, the names were easily assignable to one gender or the other but for several cases the profile picture was needed to identify the gender. In a handful of instances, it was necessary to click on the creator profile and read their description to assign a gender. While most names appeared to be first names of European or North American origin, the location of the creators was indeed not identifiable through just the Pinterest account.

#### Followers.

In terms of followers, the boards ranged between 68 and over 2.1 million, with a mean of 29,009 and a median of 512 followers. It is quite astonishing that all boards had followers and that many had a substantial number. The follower statistics suggest that these travel boards are not only public but represent travel dreams that are actively followed by others who will be notified when new pins are added. That this platform-facilitated inspiration of others is happening was evident in pins that appeared across several boards, even in this small sample of boards.

#### Pins.

As far as the actual pins are concerned, the 100 boards contained a minimum of 85 and a maximum of 11,201 pins. On average, they contained 1,098 pins, with a median of 655. Only one board had over 5,000 pins. It is evident that Pinterest users put in quite a bit of effort over time into constructing these boards and have a substantial collection of travel information available at their and other’s disposal. Interestingly, the correlation between the number of pins and the number of followers was insignificant (r = -−.048; p = .637). This is somewhat surprising as one would assume that boards with a small number of pins have a smaller number of followers and vice versa. One possible interpretation is that Pinterest users predominantly create these boards for their own benefit rather than for others.

#### Contents.

The coding of the location of the pins was quite difficult in that there is no automatic location tag and the location is rarely indicated in the picture and often not even in the pin label or associated link. Often the links did not work. Further, the pins rarely contain iconic destinations. Only one picture of the Eiffel Tower was visible in the over 600 pictures analyzed. Some pins are so generic (e.g. trees in the fog) that even after clicking through the associated link, no location could be assigned. Other pins related to multiple locations (e.g. best beaches around the world) and, thus, could not be assigned to a specific continent. Human judgment was needed in almost all instances to correctly classify the location, and even though only the top row of the boards was analyzed, this required substantial time investments. For 564 pins (93%), the location could be identified in this way.

Despite these difficulties, some interesting results emerged, even for high-level coding at the continent level. First, no pins displayed in the first row of the board related to destinations in Antarctica and five pins each were located in Africa and in the Middle East. Most surprisingly, only 9 boards displayed destinations in the Pacific, although Australia, New Zealand and the South Pacific island nations often fill the pages of travel magazines and are generally considered as highly aspirational destinations. Central & South America were only present in 11% of the boards (16 pins in total) and 19% of the boards displayed an Asian destination in their first row. Europe and North America clearly dominated the boards, with 63% of boards featuring at least one European destination and 71% showing at least one pin from North America in their top row.

While the analyzed pins represent the most recent pins for each board, the actual date the pin was added is not displayed. The platform has a new option to add date ranges to boards, but users (including the author) do not seem to limit their “Places I’d Like to Go” boards to specific time periods, confirming the ongoing nature of their efforts. Thus, while there is some indication that recent pins do not include many exotic destinations, a direct link to the COVID-19 pandemic cannot be made. Only two pins directly spoke to travel restrictions: one featured the best virtual tours around the world and was labeled “Can’t travel?”, while the other presented “family staycation” ideas for spring break. Most rows (56%) displayed pins from more than one continent, suggesting that the travel dreams of Pinners generally involve at least some long-haul travel.

As described in the methodology section, the coding of the contents further involved the identification of easily detectable features that speak to the types of destinations or vacations Pinners dream about. Only one board did not have any of the coded features in its top row. What stands out is that 90% of the analyzed boards display at least one image with a water-related feature. In contrast, only five of all analyzed pins contain animals. Equally rare to find were churches and temples. Surprisingly, while the “sea” portion of the “sea, sand and sun” vacation stereotype appeared prominently across the boards, only 42% of the boards displayed an image of a beach and 28% even showed snow or ice. Mountains and cityscapes were equally popular, with 39% of boards including at least one respective image. A small number of pins (27 in total) spread over 19% of the boards related to food.

Other categories that emerged but were not specifically coded include general “bucket lists” or more specific “best places in X” lists, hinting at the role of these boards in providing inspiration at various levels. Further, quite a few images related to specific hotels, restaurants or attractions, suggesting that Pinners do not just dream of destinations in the sense of geographic locations. Moreover, maps that displayed itineraries or clusters of attractions were included in several of the boards, indicating that Pinterest boards are used to archive information that directly helps with later travel planning.

### In-Depth Analysis

The four boards selected to represent the in-depth analysis display a broad range in terms of their structure, size, popularity and contents. They provide diverse content types and describe travel dreams at different levels of specificity. They illustrate what contextual information can be easily derived through qualitative analysis of boards and what insights are possible in terms of the ways different Pinners go about structuring their ongoing travel searches.

#### Katy.

This 45-year-old Pinner (the year of birth forms part of her username) lives in Texas (her 68 boards pertain to things like cowboy boots, quilts, pies, Tex Mex food, Hill Country Living and Texas Courthouses). Her profile has over 23,000 followers overall and almost 250,000 pins. Her “Places I’d Like to Go” board and a board called “Favorite Places & Spaces” are her only travel-related boards, with the latter pertaining to places she has already visited. The “Places I’d Like to Go” board has 2,727 pins and 1,300 followers. Katy clearly dreams of road trips to national parks in the United States and Canada. Her board is dominated by views of mountains, glaciers, rivers, canyons, waterfalls and empty roads winding through picturesque landscapes. Despite this focus on nature, her pins are almost completely void of animals. Katy also dreams of seeing fall colors and snow. A few stray pins pertain to beach vacations (mostly Florida and Hawaii). There are quite a few pins about destinations in Europe. Regarding more “exotic”, long-haul travel, Katy appears to dream about going to the Maldives, the U.S. Virgin Islands, Madagascar, Japan and several places in China. One of her China pins (Rainbow Mountains) is a pin that appears on many Pinners’ travel boards. Katy also pins antiquing-related destinations and likes inspirational lists like “Premade Vacation Itineraries”, “12 Awesome Oregon Coast Vacation Rentals for Less Than $100” and “5 Spots in Alberta that Will Blow Your Mind”.

#### Chloe.

A vocalist from Florida, Chloe uses Pinterest mostly to collect baking and holiday decoration ideas. Her 73 boards have over 25,000 pins and her profile is followed by more than 2000 Pinners. Chloe has four travel-related boards, although only two of them have more than 25 pins, namely her “Places I’d Like to Go” board (424 pins, 686 followers) and her “Caribbean Travel” board (72,857 pins and 33,790 followers). Chloe’s “Places I’d Like to Go” board seems to have changed about mid-way. Her earlier pins were a mix of European destinations with a few more exotic places like Jordan, Egypt and Belize. She also went through a Great Britain phase, with a cluster of pins showing British towns and Scottish castles and landscapes. Notable is also her love for European churches. Her more recent pins are almost exclusively about beaches. Featuring destinations from Fiji to Greece and the Philippines, the pins display picturesque, sandy beachscapes with palm trees, shells and sailboats in the background. Her board looks like a vision board, with motivational pins that say thinks like “Dear Beach, I think of you ALL THE TIME”. There are no maps, no itineraries, no hotel-related pins or anything else that would hint at actually planning a trip. Chloe dreams of traveling to serene, empty beaches, no matter where in the world they are.

#### Rheanna.

This African American woman in her late 30s who lives in Maryland has 6,000 followers and 40,146 pins distributed over 93 boards. She pins about a lot of things, from weight loss and fitness to gift ideas and home decorating. She has a total of 11 travel-related boards that feature destinations like Egypt, Paris, New Orleans, Thailand, Jamaica, Greece and Bali. Two of her boards pertain to the planning concrete trips. Her “Places I’d Like to Go” board has 788 pins and 361 followers and stands in stark contrast to Chloe’s board as it is filled with practical travel pins and bucket lists. From tips for solo female travelers, to in-flight beauty recommendations, long-haul flight survival guides, packing lists and “how to travel on a budget” and “how to take travel selfies” advice, her board is full of information that pertains to trip logistics and acquiring travel skills. While New York City and Los Angeles seem to dominate her board, the bucket lists relate to destinations all over the world, from Dubai and Paris to Thailand. While Rheanna clearly dreams of traveling the world, she also seems to have a lot of anxieties and financial constraints regarding travel.

#### Laura.

Laura is in her 40s and lives in Colorado. Her Pinterest profile is followed by 399 users and features 7,077 pins that encompass topics such as wine, beauty tips, tattoos, hairstyles and crafts. Of her 37 boards, four are travel-related, with specific ones relating to Beirut, Idaho and Sicily & Malta. Her “Places I’d Like to Go” board has 342 followers, 834 pins and 27 sections for particular destinations (Table 1). Two sections stand out because of their direct relevance to dreaming about travel: “I dream of Morocco” and “Travel Dreams”. The Morocco section has pins with itineraries, lists of “hidden gems”, restaurants, accommodation, and tips for women. It also has several pins that are clearly not related to Morocco, confirming the data quality concerns raised earlier. The “Travel Dreams” section features pins that speak of “breathtaking”, “stunning”, “ultimate”, “fairytale”, “awesome”, “most creative” and “Instagram-best” destinations all over the world.

**Table 1.** Laura’s “Places I’d Like to Go” Board Sections.

Section title	#Pins	Section Title	#Pins
Nicaragua	1	Kona Hawaii	12
Guatemala	3	Stateside	17
Girls Trip	3	Spain + Portugal + Chefchaouen	31
Chile	2	Italy	41
Madagascar & Seychelles	4	Netherlands and Belgium	76
Amalfi	2	Turkey	172
Balkans	11	Sicily	44
Sri Lanka travel	6	Paris trip	8
Lebanon	2	Malta	43
Tahoe trip	4	Travel Dreams	52
France	15	Cuba Bound	53
Greece	34	I dream of Morocco	67
Peru	8	Vacation Excitement	35
Travel Wardrobe	34		

Her “Places I’d Like to Go” board only has 54 pins that are not assigned to any section. They encompass a random collection of pins that discuss when to visit different destinations, what to buy on a trip to Turkey, most iconic hotels in the world, best tapas bars in Seville, things-to-do in Kathmandu, etc.

## Discussion and Conclusion

The results demonstrate that Pinterest is used extensively to support ongoing travel-information search and that its role ranges from visual inspiration and motivation to archiving of materials to inform future trip planning efforts. The findings also hint at important patterns in pinning behavior that could be used to predict shifts in travel preferences. They further elaborate on data constraints while at the same time pointing to vast opportunities for using the platform to explore travel-related research questions.

### Theoretical and Methodological Implications

Given the almost exclusive focus in the literature on trip planning, this research provides important insights on how potential travelers conceptualize their dream vacations and engage in ongoing search to inspire their future trips. Importantly, the visual aesthetics apparent in the boards - with tranquil waters, towering mountains, icy landscapes and picturesque European cities capturing the imagination of Pinterest users - highlight the importance of more than functional needs in travel information search [26]. In addition, the research demonstrates that a platform like Pinterest that is dominated by female users can also provide a gendered perspective on travel information search that is still largely missing from the literature [27]. Especially the extensive and ongoing labor that these female travelers invest in curating these boards needs to be recognized and further investigated. The research also adds to the literature on the importance of visuals for women when planning activities [28]. Last, the findings challenge the prevailing perspectives of information search as the activity of individuals or small travel groups and highlights the collective nature and shared inspiration that Pinterest facilitates.

From a methodological perspective, the value of this research lies in identifying the richness of publicly available data on Pinterest, especially in relation to travel. The findings suggest that this information could and should be mined in different ways but also explain why it might not be easy to capture and analyze this data in automated ways. The display algorithm is opaque, the structure of boards is inconsistent, pins can include different modalities of content, the pre-classification of pins through the boards is far from perfect and the data capture would have to scrape the actual images rather than relying on capturing links. The results also call for progress in the automated analysis of images given their centrality in travel and tourism. At the same time, the research demonstrated the strength of a netnographic approach in terms of its flexibility as well as the ability to obtain rich contextual understanding. This was particularly important for understanding who the creators of these boards are, as little information beyond their gender can be extracted from the platform. Kozinets [21] writes that Pinterest netnographies are surprisingly rare and this research therefore contributes to the literature on netnographic studies relating to the platform in significant ways.

### Practical Implications

While the research was only exploratory, it offers some important practical implications for tourism marketing. The fact that identifying the location of the pins was problematic constitutes a huge problem for destination marketers and emphasizes the need to feed the Internet, and especially social media, with visuals that have captions and to optimize website and social media contents for pinning. In addition, the vacation dreams represented by these boards allow for understanding of the competitive positioning of destinations in the minds of potential travelers. However, not being able to easily identify the location of the pins or of the Pinners is a big issue from a practical point of view. Similarly, the continued dominance of North American and European users limits the insights that can be derived from Pinterest on a global scale.

The findings also suggest that visuals are critical in the dreaming phase. Some pins are added because of their aesthetic qualities, without any indication of the location. This has important implications for recommender system design in that it stresses the relevance of visual recommendations and the need for systems that support the search for inspiration [29]. At the same time, the results also demonstrate the huge potential of Pinterest boards to be mined for recommender system development as they represent elaborate user profiles and categories of dream destinations.

### Limitations and Future Research

Netnography offers three distinct data collection operations, two of which were applied to this research. While it is common to leave out the interaction movement in netnographic tourism research [22], future research should consider eliciting research from Pinterest users who use the platform for ongoing travel information search to obtain further contextual data about the how’s and why’s of pinning travel-related contents. Pinterest has a messaging function that makes contacting specific pinners possible. Of course, such data collection will require different ethical considerations.

The current research only analyzed snapshots of boards. Longitudinal analysis of specific boards would allow for additional insights regarding pinning frequency and patterns. Also, this research did not code the source of the pin (e.g. repins from other Pinterest users, pins from social media or pins from websites). Such data could provide important understandings of Pinterest use behaviors as well as offer information about the sources of inspiration for ongoing travel information searchers. Finally, the content analysis of the types of destinations/attractions included in the boards was rudimentary and mainly for illustrative purposes. Many other aspects of these images could be coded manually or using machine learning approaches to identify what kinds of images/contents Pinners find worthy of inclusion in their travel-related boards. Co-occurrences of destinations could be explored using network analytic approaches.
